# Highly fluorescent few atoms silver nanoclusters with strong photocatalytic activity synthesized by ultrashort light pulses

**DOI:** 10.1038/s41598-020-64773-z

**Published:** 2020-05-19

**Authors:** Jesica M. J. Santillán, David Muñetón Arboleda, Diego Muraca, Daniel C. Schinca, Lucía B. Scaffardi

**Affiliations:** 1 Centro de Investigaciones Ópticas (CIOp), (CONICET - CIC - UNLP), Camino Centenario y 506, 1897 Gonnet, La Plata, Argentina; 2 Instituto de Investigaciones Fisicoquímicas Teóricas y Aplicadas, INIFTA (CONICET-UNLP), Diagonal 113y 64, La Plata, Argentina; 3 Instituto de Física “Gleb Wataghin”, Universidade Estadual de Campinas, Campinas, Brazil; 4 Facultad de Ingeniería, UNLP, 115 y 49, 1900 La Plata, Buenos Aires, Argentina

**Keywords:** Synthesis and processing, Synthesis and processing

## Abstract

While there are conventional chemical synthesis methods to generate metal nanoclusters (NCs), many of them are adversely affected by the unavoidable contamination of the nanoproduct solution, resulting in aggregation, background noise in analytical chemistry, toxicity, and deactivation of the catalyst. In this work, physical method of ultrafast laser ablation as a “green” synthesis approach together with mechanical centrifugation to obtain silver NCs, simplifying widely the chemical synthesis requirements, is proposed. Remarkably, compared with conventional methods for synthesizing Ag NCs, this new approach starts with a colloid that contains nanosized particles as well as smaller species, managing to obtain colloids with few atoms NCs by centrifugation. Those colloids were analyzed by fluorescence spectroscopy observing UV bands corresponding with HOMO-LUMO cluster transitions. Besides, independent HRTEM measurements were made confirming the presence of few atoms Ag NCs, as well as small NPs in different formation stages. Equally important, photocatalytic efficiency of the obtained NCs was studied through degradation of Methylene Blue (MB) when it was mixed with as-prepared or highly centrifuged colloid, showing an enhanced photocatalytic efficiency of 79% as compared to 57% for pure MB after 180 min of illumination. Consequently, this work contributes to establishing a simple approach to synthesize highly fluorescent and photocatalytic NCs.

## Introduction

Metal clusters are known as few nanometer sized particles made up of subunits which can be atoms of a single element (mono metal), or of several elements (alloys). Their novel chemical and physical properties are dependent only on the number of atoms they contain. These size-dependent properties, which make them suitable for applications in catalysis^1^, photoluminescence^2^, biomedical^3^ and magnetism^4^, among others, show significant deviations from their bulk and large nanoparticles (NPs) counterparts. For this reason metal clusters may be considered as new materials covering the intermediate stage between single atoms/molecules and bulk materials. There are different procedures for clusters synthesis, which rely on the use of microemulsions^5,6^, vesicles^7^ and electrochemistry techniques^4,8,9^, thiol cappings^10^ and other ligand-protected gold, silver, copper and bimetal/alloy NCs^11,12^ synthesized by wet chemistry. Wu *et al*.^13^ presented a review on directed self-assembly (DSA) of ultrasmall (sub-two-nanometer regime) metal NCs through wet chemistry methods. They show that surface ligands are important for self-assembly processes for the different noble metals reviewed. Gold NCs have been studied for their potential use in nanosensors, bioimaging and as biomarkers, the latter based on its well-known biocompatibility characteristics. Copper NCs were studied during the last years for applications in chemical sensors and biolabeling agents based on their catalytic and optical properties. Particularly, DSA allows bright emission in the blue-green for nanoribbons assembled architectures^13^. However, they have comparative low quantum yield with respect to other noble metal NCs.

On the other hand, ligand-protected Ag NCs have received much attention as novel fluorophores due to their good photostability, high quantum yield emitters and low toxicity. These properties make them suitable for microscopy settings, with potential biocompatibility and applications to sensoring and bio-labelling when DNA is used as template^14–16^. Selva *et al*.^17^ showed the electrocatalytic properties of 2–3 atoms Ag clusters for the oxidation of ethanol and other alcohols to provide protection against ethanol toxicity in cultured mammalian cells. Harb *et al*.^18^ studied theoretical and experimentally, the optical absorption of small Ag clusters in an Ar matrix, analyzing the influence of *s* and *d*-electrons in the absorption spectra as the number of atoms increases. Zheng *et al*.^19^ synthesized Ag clusters of 2 nm average diameters in sodium dodecyl sulfonate (SDS) as a protective agent, observing plasmon and cluster peaks in absorption spectra. Chemical synthesis methods used in these works tend to yield highly monodisperse clusters suspensions, although mixed with unwanted chemical precursors, which often leads to purification steps to remove the chemical by-products and may derive in expensive and complicated procedures. For these reasons, we propose to find a route to synthesize Ag NCs free of by-products to boost their catalytic and biomedical properties.

Currently, development of green processes for synthesis of noble metal NPs has become an active branch of nanotechnology^20^. Pulsed laser ablation in liquids has emerged as an alternative technique for synthesize metal NPs since nanomaterials may be generated from almost any solid sample with high purity. Since laser ablation process produces ions and atoms in the plasma plume that reach different nucleation stages, large (radii $>$ 20  nm), medium (2 nm $<$ radii $<$ 20  nm), small (1 nm $<$ radii $<$ 2  nm) and very small (radii $<$ 1  nm) NPs are formed^21–24^.

For potential catalysis application, it is important to produce small and very small clusters, since they seem to be the most active catalyst sites. Reactivity is highly dependent on their electronic structure, leading to large variations even for sizes differing by only single atoms. In particular, Ag NCs are known to catalyze a wide range of reactions. Although the exact catalytic mechanisms remain a subject of debate, the reactivity and catalytic properties of Ag NCs depends on several factors such as preparation method, size, morphology and oxidation state of the cluster, supporting material, etc.

In this paper we report and analyze for the first time the synthesis of free-standing, ligand-free, stable few atoms Ag NCs obtained by femtosecond (fs) pulsed laser ablation of Ag target in water. UV-visible fluorescence emission was used to evidence and characterize the HOMO-LUMO energy band gap of few atoms NCs in the obtained Ag colloids. Since these few atoms NCs coexist with small Ag NPs of radius smaller than 2 nm, centrifugation of the as-prepared colloids with different speeds and times was used to obtain a rich population of NCs in the supernatant of the centrifuged colloids. A careful work with High Resolution Transmission Electron Microscopy (HRTEM) performed on this supernatant evidenced the presence of very small Ag NCs.

To assess the photocatalytic activity of NCs, degradation of methylene blue (MB) aqueous solution mixed with the as-prepared colloidal suspension and also with the supernatant of the centrifuged colloids was analyzed while the sample was illuminated with white light. Degradation efficiency was evaluated by measuring the absorbance of MB at 660 nm at different times during illumination after addition of Ag colloids. We found that silver colloids with larger centrifugation times had stronger photocatalytic activity, suggesting that small Ag NCs present in the colloidal suspension are responsible for this enhancement. It was found that even small concentrations of Ag NCs had a stronger photocatalytic action than that reported by other authors in the literature^25–27^.

## Experimental section

Samples were obtained by 120 fs pulsed laser ablation of a 10 mm diameter and 1 mm thick high purity grade Ag solid disk target immersed in 2 ${{\rm{cm}}}^{3}$ of Milli-Q water. A liquid column of 1 cm height over the target within the vessel was left to enable obtaining high concentrated suspensions.

To separate small clusters from the large Ag NPs, the obtained colloids were centrifuged varying independently centrifugation speed and time. Absorbance and fluorescence spectra from the as-prepared colloids and supernatant of the centrifuged colloids were respectively obtained using a Shimadzu UV-1650PC spectrophotometer (200 nm to 1100 nm wavelength range) and a Shimadzu RF-5301PC spectrofluorophotometer (200 nm to 700 nm wavelength range), using for the latter an excitation wavelength ${\lambda }_{{\rm{exc}}}\,=$ 220  nm. Absorbance of MB at 660 nm during photocatalytic experiments was also performed using the same UV-vis spectrophotometer.

Morphology, cluster size and particle size distribution were analyzed by transmission electron microscopy in diffraction and phase contrast modes using a TEM-FEG (JEM 2100F) and a double-corrected Titan Themis TEM/STEM at the Brazilian Nanotechnology National Laboratory (LNNano/CNPEM, Campinas-SP, Brazil). High-angle annular dark field STEM (HAADF-STEM) images were acquired with the Titan TEM operated at 300 kV. For all the microscopy measurements, the samples were prepared few hours before the experiment by drying a drop of the colloidal dispersion on ultrathin carbon film supported on Ted Pella holey carbon copper grid.

We have tried to study the oxidation state of silver atoms in the silver NCs using the traditional XPS technique. However, due to the low concentration of NCs in our samples obtained by laser ablation and post centrifugation (estimated in less than 1 μM), compared with the 1 mM concentration of wet chemistry methods^28^, no reliable results were obtained due to the fact that we could not attain the minimum mM range value required by XPS technique. On the other hand, it is not possible to increase the concentration of NCs with ablation in the needed amount to overcome the almost three orders of magnitude difference.

Comparative experiments on photocatalytic activity with MB in presence of as-prepared Ag colloidal suspension as well as with colloids obtained after 1000 min of centrifugation were conducted. Degradation of MB under white light lamp irradiation was analyzed by recording MB absorbance at 660 nm every 20 min during 3 h. The total integrated power of the lamp in the wavelength range 430 nm to 680 nm was 0.015 W ${{\rm{cm}}}^{-2}$.

Degradation of mixtures of MB with Ag colloidal suspensions centrifuged at different times were compared against that of pure freshly prepared MB (0.04 mM), which was taken as reference. Specifically, two samples were analyzed: sample 1 (S1), prepared by mixing 0.5 mL of as-prepared Ag colloidal suspension (1.021 $\times {10}^{-3}$ mM) with 1.5 mL of MB, and sample 2 (S2), prepared by adding 1.0 mL of Ag colloidal suspension (2.075 $\times {10}^{-4}$ mM, with 1000 min centrifuged time) to 1.0 mL of MB. The mentioned mixtures in these samples were selected so as to work with absorbances from 0.05 up to 0.35. This allowed an appropriate absorbance dynamic range for a reliable degradation measurement.

The degradation efficiency of MB was calculated based on following expression:

1 $${\rm{Degradation}}\,{\rm{efficiency}}\,( \% )=100\times ({{A}}_{O}-{{A}}_{{t}})/{{A}}_{{O}}$$

where *A*_*O*_ represents the maximum dye absorption before illumination and *A*_*t*_ its maximum absorption for different illumination times.

## Results and discussions

### Fluorescence and extinction spectroscopy analysis

As it was briefly stated in the Introduction, metal NCs consist of few to tens of metal atoms. As their size approaches the Fermi wavelength of electrons (approximately 1 nm), the energy level scheme of the NCs resemble discrete levels much like molecules than solids metals. This fact leads to the observation of attractive properties like strong band fluorescence in the optical regime. Silver colloids obtained by femtosecond laser ablation present, in general, small and medium sized NPs (2 nm to 20 nm in radius) coexisting with small and very small clusters, as observed from the HRTEM images that will be shown below. In contrast to NPs, silver metal clusters comprising N $\le$ 20 atoms exhibit fluorescent transitions in the UV rather than the characteristic plasmon band in the visible^8,14^. The electron dynamics of few atoms metal structures has been studied on the basis of a physical model called jellium model^29,30^. This model is based on a low level approach that describes the valence electrons of the NCs as a free gas with negative charge moving in a weakly attractive mean field created by the spherically symmetric 3D harmonic potential of the ionic positively charged cores. In a series of fluorescence studies of water-solved Au and Ag clusters, Zheng *et al*.^31^ directly correlated the HOMO-LUMO gap of clusters to the emission energy of Au clusters and demonstrated that this transition energy follows the scaling relationship, ${E}_{g}={E}_{F}.{N}^{-1/3}$ ^32,33^ predicted by the jellium model, where ${E}_{g}$ is the energy band gap, ${E}_{F}\,$is the Fermi energy and N is the number of atoms in the cluster. This simple approach is valid in principle to describe clusters from 20 to about 1000 atoms. The match between the theoretical and experimental data sets is acceptable, even for NCs sizes below the range of intended validity of jellium approach (N $=$ 20). Moreover, the expected shell structure reproduces the magic numbers found experimentally for noble metal clusters N = 2, 8, 20, 34, 40, 58, 92^34^.

**Figure 1:**
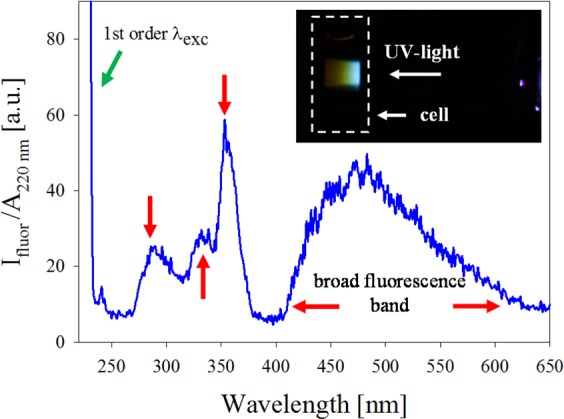
Fluorescence spectra in the UV-visible region showing isolated peaks (284 nm, 330 nm and 358 nm) and wide band between 400 nm and 625 nm corresponding to few atoms NCs. Inset shows photograph of the fluorescence induced by UV light excitation on the sample cell.

To analyze the possible presence of few atoms clusters in as-prepared silver colloids, UV-vis fluorescence spectroscopy experiments were conducted using ${\lambda }_{{\rm{exc}}}\,=$ 220  nm. To correctly asses the spectra, each one was divided by its absorbance value at ${\rm{\lambda }}=$ 220 nm (${A}_{220{\rm{nm}}}$). Figure 1 shows a typical ${I}_{{\rm{fluor}}}/{A}_{220{\rm{nm}}}$ for the as-prepared suspension. Three peaks around ${\lambda }_{{\rm{fluor}}}\,=$ 284 nm, 330 nm and 358 nm (red arrows) as well as a broad fluorescence band spanning from 400 nm to 625 nm are observed, suggesting the presence of few atoms NCs, as predicted by the jellium model. The peak out of scale corresponds to the excitation wavelength.

The bandgap energy ${E}_{g}$ may be estimated from the peak wavelength of the experimental fluorescence UV-visible bands. Considering ${E}_{F}=$ 5.49 eV for silver, qualitative information about few atoms NCs conformation may be obtained. Given these results, it is possible to obtain NCs with less than 5 atoms. From the above relation, it is clear that, for increasing N, the band peaks start to overlap with each other, giving rise to a wide band. However, this is a gross estimation and, to corroborate it, further specific experiments are needed, which, given the nature of our samples cannot be run, as will be discussed below.

Inset in Fig. 1 is a 30 s exposure photograph of the fluorescence induced by ${\lambda }_{{\rm{exc}}}\,=$ 220  nm UV light excitation on a cell containing as-prepared colloid. It can be seen a blue-reddish fluorescence, which corresponds to the wide fluorescence band centered around 480 nm.

**Figure 2:**
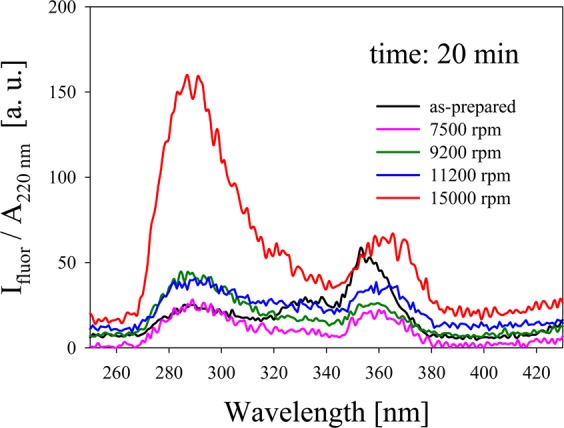
${I}_{{\rm{fluor}}}/{A}_{220{\rm{nm}}}$ for different centrifugation speeds.

As stated before, one way of separating the different species in the as-prepared colloid is by mechanical centrifugation of the sample. It was readily observed that, even for short centrifugation times and low speeds, the wide band disappeared from the supernatant spectrum, suggesting that few atoms clusters were effectively separated from the larger ones. To analyze the optimal centrifugation conditions for obtaining few atoms clusters, fluorescence of a sample synthesized by 600 μJ pulse energy fs laser ablation, was studied in the range 250 nm to 430 nm at different centrifugation speeds for a fixed centrifugation time. Since the clusters may have different absorption at ${\lambda }_{{\rm{exc}}}\,=$ 200 nm, each spectrum was divided by its absorbance value at this excitation wavelength. The ratio ${I}_{{\rm{fluor}}}/{A}_{220{\rm{nm}}}$ allows a better comparison between the spectra, as can be seen in Fig. 2.

In the fresh colloid prepared by laser ablation (without centrifugation), bands located at about 284 nm, 330 nm and 360 nm are present, associated to different Ag NCs sizes. As centrifugation speed increases, initial fluorescence bands in 284 nm and 330 nm begin to overlap forming a wide fluorescence band whose maximum is around 284 nm. For 15000 rpm, there is a sudden increase in the intensity of the latter band with respect to the 330 nm band. The interpretation of this finding will be discussed later. We will use 15000 rpm for the following analyses with the aim of studying very few atoms NCs in catalysis.

**Figure 3:**
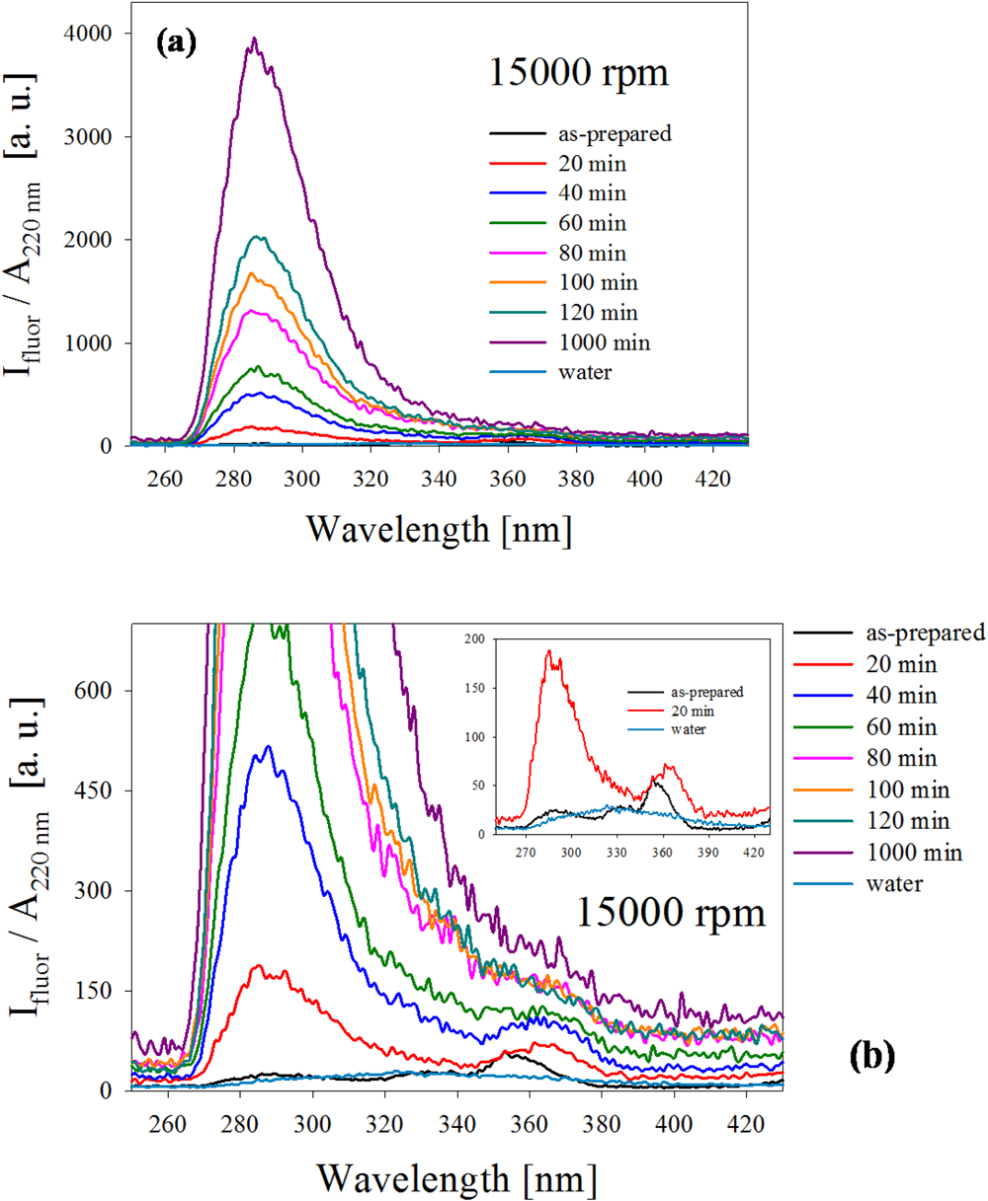
(**a**) Fluorescence spectra of colloid obtained by fs laser ablation of Ag solid target in water for different centrifugation times. Excitation wavelength is ${\lambda }_{{\rm{exc}}}\,=$ 220  nm. A dominant band at 284 nm is readily seen. (**b**) Enlargement of the fluorescence curves for as-prepared and centrifuged samples for times smaller than 120 min, showing other fluorescence bands at 330 nm and 360 nm. The bands located at 284 nm and 360 nm correspond to different few atoms Ag NCs sizes. Inset shows spectra of the as-prepared and 20 min centrifugation samples as well as that for pure water for comparison.

To assess the efficiency of centrifugation time in the separation process, the UV fluorescence properties of small Ag NCs was analyzed for 15000 rpm and different times. Figure 3 shows the spectra for 20, 40, 60, 80, 100, 120 and 1000 min centrifugation times together with the as-prepared sample. Again, the fluorescence curves were divided by the absorbance of the corresponding samples at the excitation wavelength ${\lambda }_{{\rm{exc}}}\,=$ 220  nm. In panel (a), a dominant band at about 284 nm is readily seen, which monotonically increases as centrifugation time increases. To analyze the details of this increase, panel (b) is an enlargement of the fluorescence curves for times less than 200 min, where the spectra of the as-prepared sample and of pure water are also included for comparison. It can be observed that the as-obtained sample shows the three characteristics bands centered at about 284 nm, 330 nm and 360 nm. Upon centrifugation, these fluorescence bands progressively increase, with a noticeable predominance of 284 nm band. This intensity relation reverses almost threefold after 20 min centrifugation time, as shown in the inset of panel (b), which shows spectra of the as-prepared and 20 min centrifugation samples as well as that for pure water. It can be seen that water has a negligible contribution to total fluorescence in that wavelength region. The general results of this experiment suggest that centrifugation process allows an efficient separation of few atoms clusters from those larger ones for catalysis purposes.

Recently, aggregation-induced emission enhancement (AIEE) has been proposed as a way for fluorescent enhancement of atomic systems. AIEE is a photo-physical phenomenon wherein non-luminescent or weakly luminescent molecules become highly luminescent upon the aggregation process in either poor solvents, or the solid state. Recent studies show that the restriction of intramolecular motion (RIM) is responsible for the AIEE phenomenon of these molecular rotor systems^35–37^. In general, the AIEE active molecules consist of a number of rotors, which can rotate or vibrate freely in dilute solution. However, rotations and vibrations of these rotors in the aggregated state are largely restricted, leading to the strong AIEE effect. The Xie group was the first to introduce the AIEE strategy into the nanocluster field, to prepare highly luminescent nanoclusters and cluster-based materials^38^. For the case of noble metal thiolated-NCs, AIEE is a valid mechanism for luminescence enhancement, since aggregation restricts thiol molecular motion.

However, in our case, Ag NCs are synthesized in a ligand-free, pure water environment. This fact, together with the fact that luminiscence measurements were made in a diluted solution, precludes AIEE as the dominant fluorescence mechanism. Besides, the Ag NC fluorescence band at ${\lambda }_{{\rm{fluor}}}=$ 284  nm exhibit no shift after long centrifugation times. This suggests that Ag NCs luminescence arises from HOMO-LUMO molecular-like transitions, as derived from the jellium model^39^.

**Figure 4:**
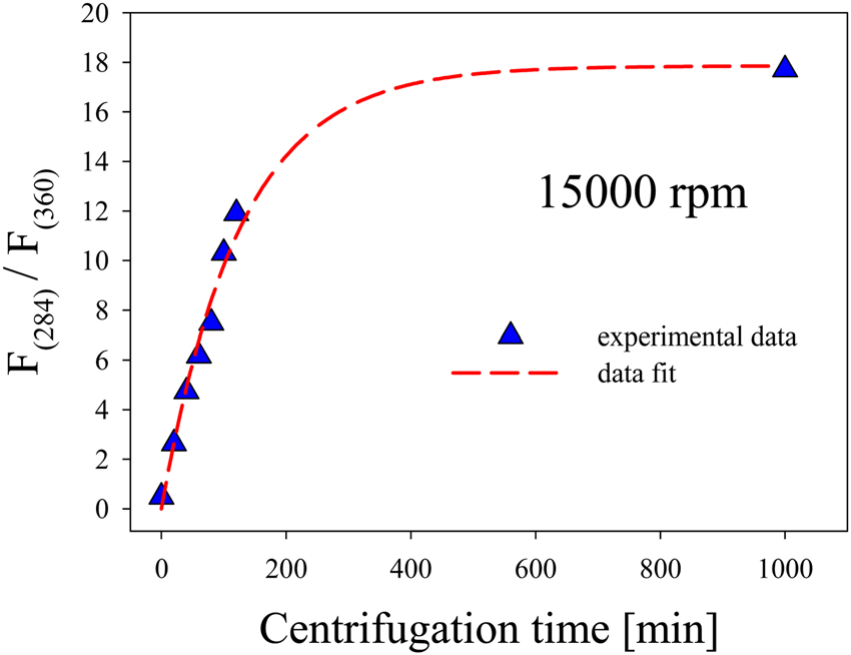
Relationship between the fluorescence intensity of 284 nm and 360 nm bands as a function of centrifugation time. A plateau at ${F}_{(284)}/{F}_{(360)}\,=$ 18 is reached after 500 min centrifugation time.

Figure 4 shows the ratio between the fluorescence intensity of 284 nm and 360 nm bands for a given centrifugation speed (15000 rpm). Dashed curve is a mathematical fit of the data points suggesting that centrifugation is an effective mechanism to isolate smaller few atoms NCs present in a colloid sample synthesized by laser ablation. It can be seen that even with small centrifugation times, it is possible to have a ratio larger than unity. On the other hand, it also allows obtaining a given relative amount of the smaller few atoms Ag NCs by an appropriate selection of centrifugation time. From this centrifugation speed, a plateau at about ${F}_{(284)}/{F}_{(360)}=$ 18 is reached after 500 min, suggesting an appropriate time to obtain the highest concentration of the smaller few atoms Ag NCs from the initial colloid.

**Figure 5:**
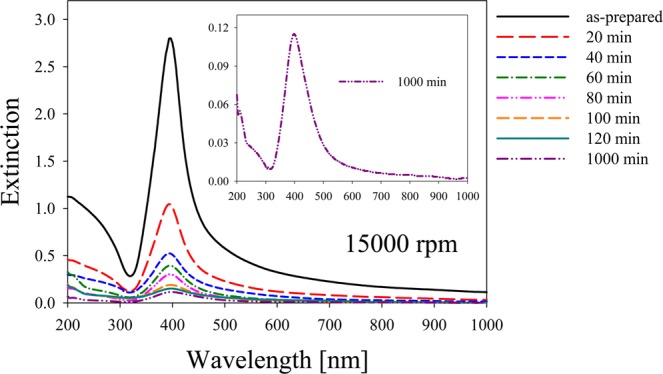
Absorbance spectra of supernatant fs laser ablation Ag colloids for as-prepared sample and different centrifugation times. Inset depicts the absorption spectrum of the colloid centrifuged for 1000 min appropriately rescaled to show the bands related to the presence of clusters of few atoms in the range 200 nm – 300 nm.

Optical absorbance spectra of the same supernatant samples used for fluorescence studies were also recorded. Figure 5 shows these spectra, where the as-prepared sample presents the characteristic Ag NPs plasmon at 400 nm, together with an intense absorbance feature in the range 200 nm to 300 nm, which corresponds to a superposition of silver interband transitions^40^ and to the presence of NCs. Inset in Fig. 5 depicts the absorption spectrum of the colloid centrifuged for 1000 min appropriately rescaled to show the bands in the range 200–300 nm due to the presence of clusters of few atoms (N $<$ 10 atoms), such as reported by Ledo-Suárez *et al*.^41^. Presence of the Ag NPs plasmon peak in the absorbance spectra for all samples, together with the observed NCs fluorescence (Fig. 3) indicate that both species coexist in the colloids. This fact denotes that NPs cannot be fully removed during centrifugation process.

The interband transitions region of Ag NPs also coincides with the HOMO-LUMO fluorescence of Ag NCs. For samples subjected to increasing centrifugation times, plasmon absorption band becomes smaller and wider, suggesting that the supernatant present a smaller number of NPs with radius smaller than 1 nm. The absorption of interband transitions flattens almost to zero for large centrifugation time samples, drastically reducing the absorption of NCs fluorescence by the NPs. This quenching reduction may explain the growth in fluorescence intensity shown in Fig. 3.

**Figure 6:**
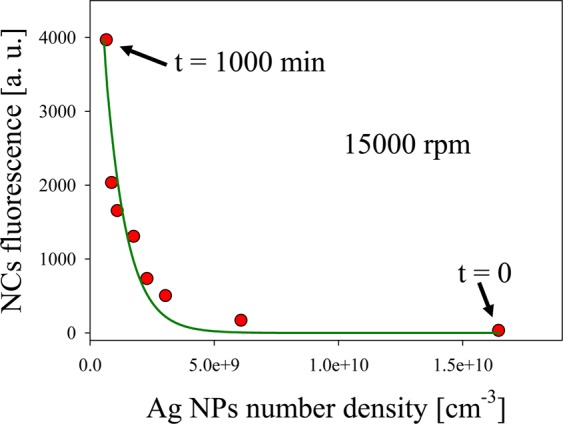
Quenching of NCs fluorescence band as a function of Ag NPs number density.

Fluorescence quenching as a function of Ag NPs number density is clearly shown in Fig. 6. For t $=$ 0  min, the number density of Ag NPs is $2.8\times {10}^{10}\,{{\rm{cm}}}^{-3}$, while for t $=$ 1000  min, is $5.8\times {10}^{8}\,{{\rm{cm}}}^{-3}$. Full circles represent fluorescence intensity for the different Ag NPs number densities, which were calculated using Lambert-Beer law at plasmon maximum for the different centrifugation times used. An exponential decay curve nicely fits experimental data taken at 15000 rpm. The decrease in fluorescence intensity with the increase in the number of Ag NPs per unit volume is due to the quenching effect produced by the absorption of NCs fluorescence bands by large NPs.

**Figure 7:**
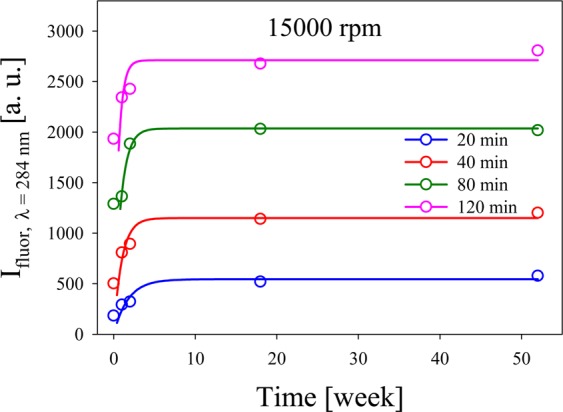
${I}_{{\rm{fluor}}}$ of Ag NCs at ${\rm{\lambda }}\,=$ 284  nm for different times.

The stability of few atoms Ag NCs over a long time was analyzed through the measurement of some fluorescence spectra shown in Fig. 3 for t $=$ 0. Figure 7 shows the fluorescence intensity of Ag NCs at ${\lambda }_{{\rm{fluor}}}\,=$ 284  nm excited with ${\lambda }_{{\rm{exc}}}\,=$ 220  nm for different times. The behavior of samples centrifuged for different times, during several weeks was studied.

It is interesting also to notice that, throughout our studies, the fluorescence peak wavelength remained unchanged from week 2 to week 52, revealing that the clusters obtained are stable for long time after centrifugation, indicating a good stability. Hollow circles are the experimental results and full lines are for guide-to-the-eye purpose.

### High resolution and corrected electron microscopy analysis

**Figure 8:**
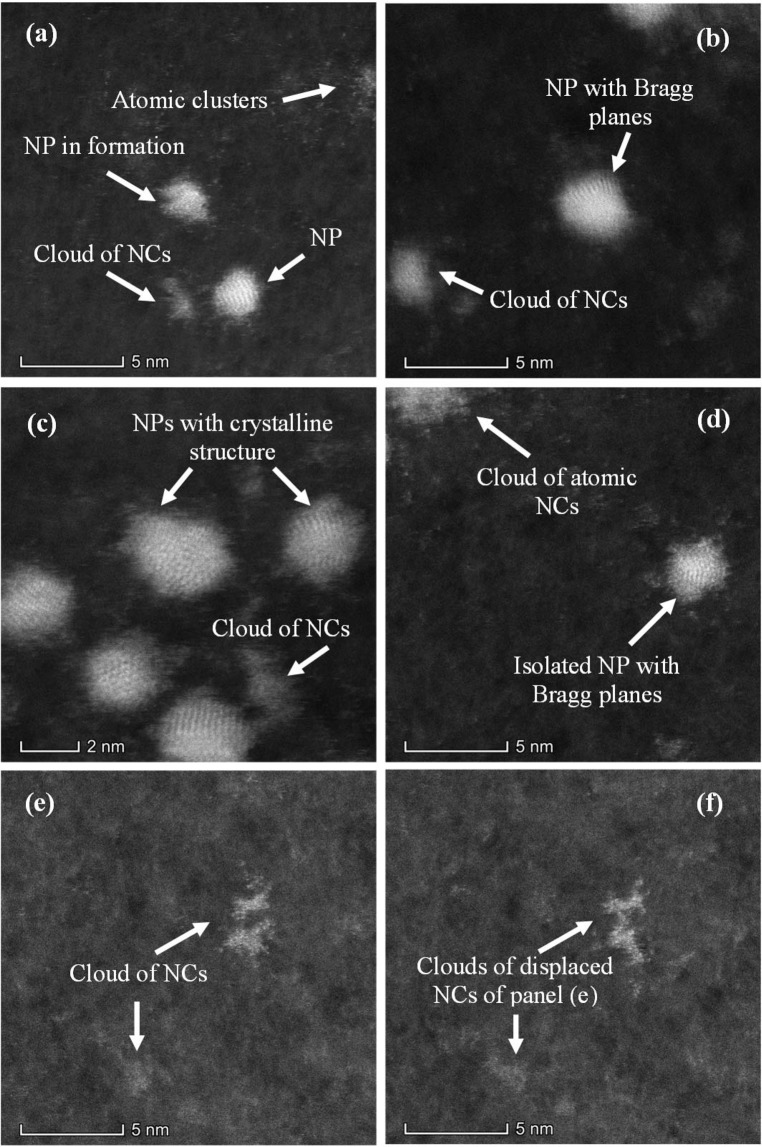
Clusters in different aggregation stages, as seen with HAADF technique. The panels show the coexistence of single NP and clouds of NCs. For details see text.

To identify morphology and structure of small clusters present in colloids, high resolution and double-corrected electron microscopy analysis was performed using HAADF-STEM mode for image quality improvement. Figure 8 shows a wide panorama of clusters in different aggregation stages. Coexistence of 1 nm radius NPs together with few atoms NCs is readily observed. At the bottom of Fig. 8(a), Ag NP of about 2.5 nm diameter shows Bragg planes evidencing crystalline structure. At the center of the image, a cluster in a proto-particle stage with crystalline structure but without a defined morphology is observed. Atomic clusters at the upper right corner of the image can also be seen. At the left of the larger NP, there is another visible atomic cluster agglomeration. At the center of Fig. 8(b), a formed NP with Bragg planes is observed, while the bright dots are atoms clusters. Moreover, clusters of atoms appear at the upper right and bottom left corner. Ag NPs of about 2 nm in size showing crystalline structure can be observed in Fig. 8(c), together with a cloud of atomic clusters in between. An isolated Ag NP showing Bragg planes can be observed in Fig. 8(d). In the upper left there is an agglomeration of atomic clusters. Clusters of individual atoms of 0.1 nm in size (almost in the limit of Titan resolution), can be observed in Fig. 8(e). Panel (f) shows the same area as Fig. 8(e), taken a few seconds later. The atoms have moved due to their interaction with the electron beam. The facts shown in Fig. 8 are in agreement with the results of the fluorescence spectra produced by atomic clusters (Figs. 1 to 4) and the plasmonic band observed in the absorbance spectra in Fig. 5.

**Figure 9:**
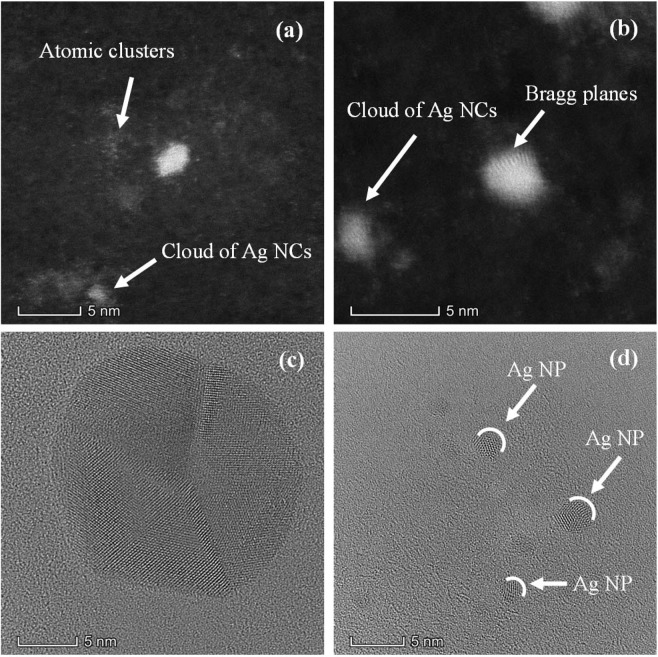
(**a**) Single Ag NP. At the bottom left corner, a cloud of Ag NCs can be observed. White dots around the Ag NP are atomic clusters. (**b**) Isolated NPs where some Bragg planes are seen. Cloud of NCs at the far left. (**c**) Bright field HRTEM showing an isolated Ag NP. Atoms can be seen in different domains. (**d**) Small Ag NPs with atoms in cluster domains.

Atomic clusters and clouds of Ag NCs can be observed in panels (a) and (b) of Fig. 9. Figure 9(b) shows Bragg planes in Ag NP of 1.5 nm radii, evidencing a crystalline structure. Figure 9(c,d) correspond to bright field HRTEM images of isolated NPs. Three sectors exhibiting silver atoms with different Bragg plane orientations are clearly seen in Ag NP of panel (c). Figure 9(d) show several isolated Ag NPs with radii smaller than 2 nm, where it is possible to observe cluster domains with different plane orientations.

We have tried to make independent measurements of cluster mass by the traditional techniques of electrospray ionization-mass spectroscopy (ESI-MS). This technique is used for mass determination of soluble complexes or metal NCs stabilized with ligands^42–44^. However, it was not possible to use ESI for our colloidal metal NCs suspensions since they had no organic components that may act as a binding molecule. Because of this, they cannot be ionized by the electrospray and, in turn, this may damage the electrode and the equipment source. On the other hand, there is another typical technique for mass determination of clusters based on laser desorption ionization followed by mass spectrometry (LDI-MS), or matrix assisted laser desorption ionization with mass spectrometry or time of flight (MALDI-MS or MALDI-TOF). Here again, the existence of ligands is important for a correct and reliable laser desorption. Since our samples have no organic components, it was not possible to conduct a MALDI-MS or MALDI-TOF experiment.

### Photocatalytic activity

Metal NCs have been studied as catalytic systems in different types of reactions such as CO oxidation, selective oxidation, selective hydrogenation, organic reactions, electrocatalytic^17,45^, and photocatalytic reactions^11,46–51^. These processes have been mainly explored for gold NCs. Zhu *et al*.^47^ synthesized Au NCs protected by thio-β-cyclodextrin and paired with TiO_2_. They found that Au NCs would serve as electron acceptors, which could inhibit the recombination of electron-hole pairs allowing processes as organic pollutants degradation. Chen *et al*.^48^ used glutathionecapped Au NCs modified TiO_2_ films and NPs as a photosensitizer for hydrogen generation through water splitting. Besides, Chen *et al*.^49^ showed that thiol-protected gold clusters modifying mesoscopic TiO_2_ films, inject electrons into TiO_2_ under visible excitation, permitting the generation of photocurrents in a metal cluster-sensitized solar cell. On the other hand, bimetal Au-Ag NCs show changes in their electronic structure and therefore its light absorption properties. This suggests the possibility to tune them to specific catalysis^11^ and photocatalysis applications^45^. Particularly, for the case of Ag NCs, several researches have been performed regarding the organic molecules degradation through photocatalysis. Samai *et al*.^50^ showed that catalytic activity of Ag NCs supported CeO_2_ NPs was enhanced 80% in relation to CeO_2_ NPs, to degrade acridine red. El-Roz *et al*.^51^ encapsulated Ag NCs in the pores of zeolite NPs to photocatalyze formic acid to H_2_ and CO_2_ under visible light. However, to the best of our knowledge, there are no works devoted to the study of photocatalytic processes by few atoms Ag NCs generated by laser ablation without ligand cappings.

**Figure 10:**
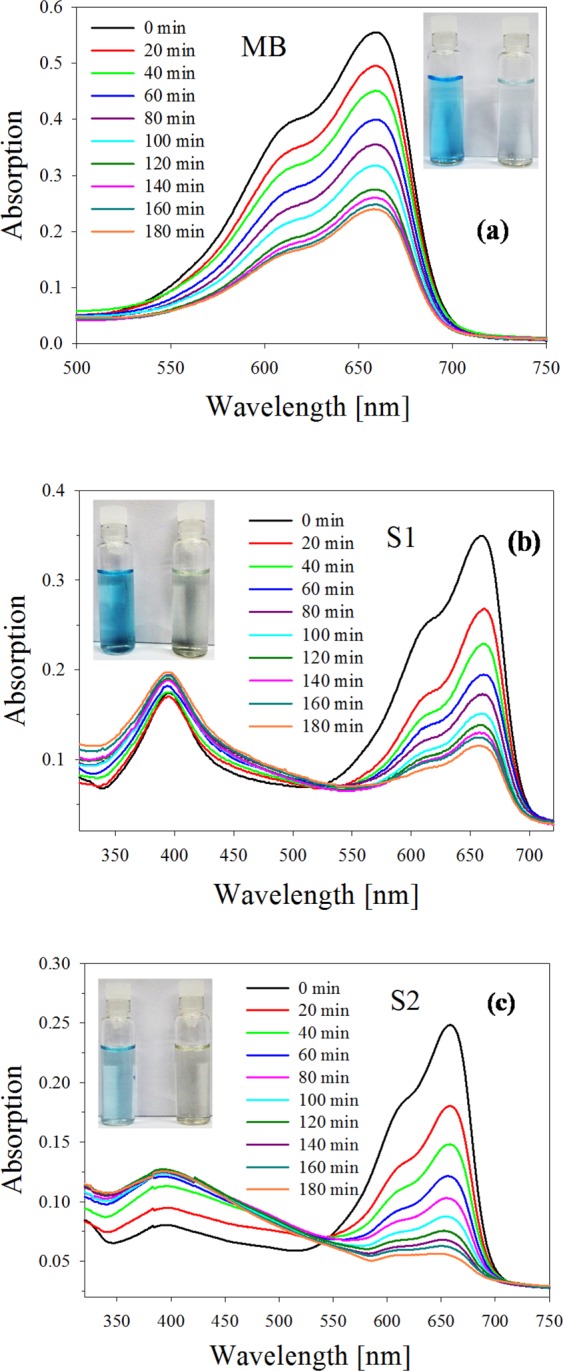
Degradation of MB. (**a**) Absorption spectra of pure MB sample illuminated by a white light lamp taken at fixed time intervals. (**b**) Absorption spectra of sample S1 taken under the same conditions as in (**a**). (**c**) Absorption spectra of sample S2 under the same conditions as in (**a**).

Reactivity of NCs is high compared to their bulk counterparts, due to their high surface to volume ratio. It is highly dependent on NCs electronic structure, which, in turn, also depends on their size. This fact may lead to large variations in reactivity even for sizes differing by only single atoms. In our case, photocatalytic activity of Ag colloidal suspension containing a synthesized NPs $+$ NCs (S1 sample) and 1000 minutes centrifuged sample (S2) containing a lesser amount of NPs and the smaller sized NCs was assessed by degradation of freshly prepared MB (0.04 mM). Figure 10(a) shows the absorption spectrum of pure MB solution at different time intervals while illuminated by a white light lamp. The main MB absorption band at 660 nm decreases gradually as exposure time elapses showing native dye photocatalytic degradation. The decrease of the absorption peak can be used to quantitatively asses the degradation through the contrast, defined by Eq. (1).

Dye degradation was easily identified by color change in the solution, from deep blue to faint light blue after 3 h exposure to white light, as can be seen in the inset of Fig. 10(a). Final degradation contrast reached a value of 57%.

Figure 10(b,c) show the spectra sequences for the case of sample S1 and S2 respectively, taken at the same time intervals as pure MB and exposed to the same white light as before. It can be seen in panel (b) that absorbance of MB at 660 nm decreases, while the characteristic silver plasmon absorbance peak at 400 nm increases as time elapses. For the case of sample S2 (Fig. 10(c)), it is seen that the absorbance at 660 nm decreases progressively faster than for the case of sample S1, while the silver plasmon absorbance increases at a lower height and widens, indicating the presence of small Ag NPs, as stated previously. Inspection of the time evolution of the absorbance band at 660 nm in Fig. 10 suggests that dye photobleaching gets stronger from pure MB to sample S2. The insets in panels (b) and (c) show the initial and final colors of the colloids of both samples, which visually agree with the quantitative decrease of the absorbance.

**Figure 11:**
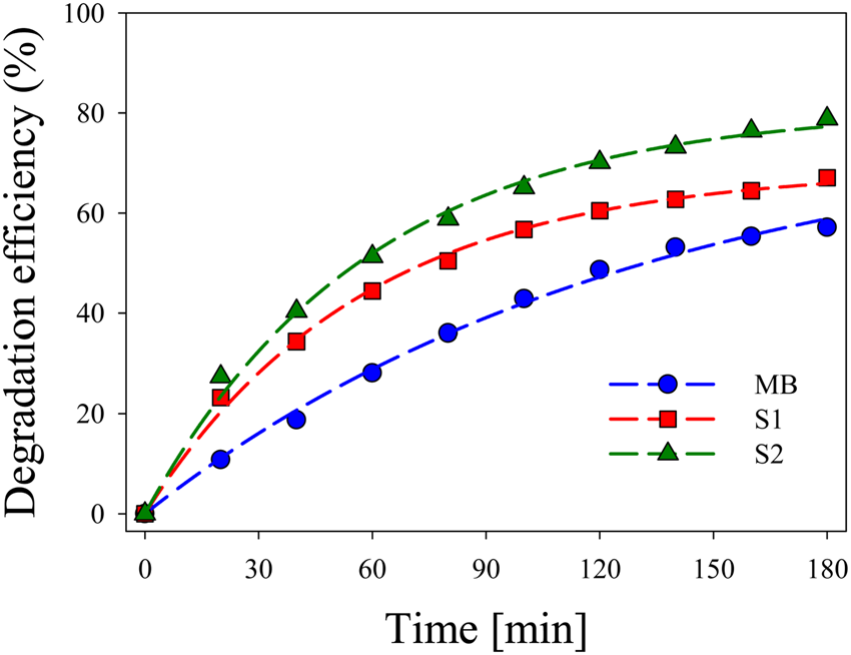
Experimental degradation efficiency for pure MB and S1 and S2 samples calculated from Fig. 10(a), (b), (c).

For a quantitative assessment of this finding, the contrast of the different spectra was calculated according to Eq. (1). Figure 11 shows the calculated values of the degradation efficiency (in %) for the two samples, besides for the pure MB. While for the latter, degradation amounts to 57% after 3 h of exposure, samples S1 and S2 show a degradation efficiency of 67% and 79% respectively for the same exposure time. These results confirm the clear photocatalytic activity of Ag NPs and NCs present in the colloidal suspension. The high degradation efficiency obtained with the as-prepared colloid used in S1 sample is due to the enhanced catalytic activity produced by the presence of Ag NPs together with few atoms NCs (as can be seen in Fig. 1(a,b)). However, sample S2, consisting mostly of smaller few atoms Ag NCs, exhibits still larger catalytic activity than S1 sample.

It is worth noticing that, despite the low concentration of clusters used in S2 sample, the above mentioned 79% degradation efficiency is obtained. This fact suggests that smaller few atoms Ag NCs have an enhanced catalytic activity may be due to their larger surface/volume ratio. The rate of degradation obtained in our system is much faster than that obtained by other authors^23^ who exposed a mixture of MB and Ag NPs to sunlight during 72 h. We obtained, at least, 67% degradation after 3 h while these authors obtained the same percentage after 66 h. Our results show a dramatic enhancement in photocatalytic activity compared with results from the literature.

## Conclusions

We report, for the first time, the synthesis of free-standing few atoms Ag NCs by fs pulse laser ablation in liquids. This process produces ions and atoms that reach different nucleation stages, producing colloids with a variety of sizes. Ag NCs were separated from their larger counterparts by centrifugation. Their presence was evidenced by fluorescence and HRTEM techniques.

UV-vis fluorescence spectroscopy experiments, conducted using ${\lambda }_{{\rm{exc}}}\,=$ 220  nm, shows three peaks around ${\lambda }_{{\rm{fluor}}}\,$located at 284 nm, 330 nm and 358 nm as well as a broad fluorescence band (not reported so far) spanning from 400 nm to 625 nm. Clusters obtained in the as-prepared colloid present the HOMO-LUMO bandgap energies (${E}_{g}$) between 2 eV and 4.4 eV. Fluorescence studies in synthesized colloids suggest that clusters with few atoms are produced with ultrashort pulse laser ablation.

When Ag NPs and NCs species are all present in an as-prepared colloid, few atoms clusters fluorescence in the range 250 nm and 300 nm is absorbed by the NPs interband transitions, resulting in low yield fluorescence. In this sense, NPs act like “quenchers” of the fluorescence. Upon centrifugation, NPs are separated from the clusters, leaving a supernatant with a smaller NPs concentration, thus decreasing clusters fluorescence quenching.

For subnanometer Ag NPs (about 180 atoms), it is possible to observe an incipient plasmonic resonance at 400 nm in their absorbance spectra^22,52^. There seems to be a region of atoms number 20 $<$ N $<$ 180, for which clusters neither fluoresce nor have plasmonic absorption. This characteristic is similar to that stated by Vázquez-Vázquez *et al*.^53^ for copper NCs.

HRTEM analysis showed the presence of clusters in different aggregation stages. Atomic cluster agglomerations, together with clusters in a proto-particle stage with crystalline structure but without a defined morphology were observed. Other scanned regions of the sample show clusters of different sizes from 0.1 nm up to 1.5 nm radius NP with Bragg planes. Also, many clouds of free-standing Ag NCs were observed in different parts of the samples.

Photocatalytic activity of Ag colloidal suspension was assessed by degradation of freshly prepared MB. Samples containing NPs $+$ NCs (S1) and mainly small NCs (S2) added to MB were exposed for 3 h to a white light lamp. Degradation efficiency of 67% and 79% for samples S1 and S2 respectively were obtained after this exposure time. These results suggest that small Ag NCs present in sample S2 show higher photocatalytic activity may be due to their large surface/volume ratio.
